# Characterizing the Biology of Lytic Bacteriophage vB_EaeM_φEap-3 Infecting Multidrug-Resistant *Enterobacter aerogenes*

**DOI:** 10.3389/fmicb.2019.00420

**Published:** 2019-03-05

**Authors:** Jiangtao Zhao, Zheng Zhang, Changyu Tian, Xiao Chen, Lingfei Hu, Xiao Wei, Huan Li, Weishi Lin, Aimin Jiang, Ruo Feng, Jing Yuan, Zhe Yin, Xiangna Zhao

**Affiliations:** ^1^Department of Histology and Embryology, School of Basic Medical Sciences, Zhengzhou University, Zhengzhou, China; ^2^College of Food Science, South China Agricultural University, Guangzhou, China; ^3^Institute of Disease Control and Prevention, Chinese People’s Liberation Army (PLA), Beijing, China; ^4^State Key Laboratory of Pathogen and Biosecurity, Beijing Institute of Microbiology and Epidemiology, Beijing, China

**Keywords:** *E. aerogenes*, bacteriophage, vB_EaeM_φEap-3, genome sequencing, *Myoviridae*

## Abstract

Carbapenem-resistant *Enterobacter aerogenes* strains are a major clinical problem because of the lack of effective alternative antibiotics. However, viruses that lyze bacteria, called bacteriophages, have potential therapeutic applications in the control of antibiotic-resistant bacteria. In the present study, a lytic bacteriophage specific for *E. aerogenes* isolates, designated vB_EaeM_φEap-3, was characterized. Based on transmission electron microscopy analysis, phage vB_EaeM_φEap-3 was classified as a member of the family *Myoviridae* (order, *Caudovirales*). Host range determination revealed that vB_EaeM_φEap-3 lyzed 18 of the 28 *E. aerogenes* strains tested, while a one-step growth curve showed a short latent period and a moderate burst size. The stability of vB_EaeM_φEap-3 at various temperatures and pH levels was also examined. Genomic sequencing and bioinformatics analysis revealed that vB_EaeM_φEap-3 has a 175,814-bp double-stranded DNA genome that does not contain any genes considered undesirable for the development of therapeutics (e.g., antibiotic resistance genes, toxin-encoding genes, integrase). The phage genome contained 278 putative protein-coding genes and one tRNA gene, tRNA-Met (AUG). Phylogenetic analysis based on large terminase subunit and major capsid protein sequences suggested that vB_EaeM_φEap-3 belongs to novel genus “Kp15 virus” within the T4-like virus subfamily. Based on host range, genomic, and physiological parameters, we propose that phage vB_EaeM_φEap-3 is a suitable candidate for phage therapy applications.

## Introduction

Over the last three decades, *Enterobacter aerogenes* has increasingly been recognized as an important opportunistic and multidrug-resistant bacterial pathogen associated with nosocomial infections (Davin-Regli and Pages, 2015). The more frequent reports of carbapenem-resistant *E. aerogenes* are particularly concerning from a public health standpoint. Carbapenems are first-line drugs for the treatment of severe nosocomial infections caused by multidrug-resistant Enterobacteriaceae (Qin et al., 2014). Owing to the emergence of carbapenem-resistant strains, treatment options for patients suffering from *E. aerogenes* infection are limited, which can have serious consequences. As such, clinicians should be alert to carbapenem-resistant *E. aerogenes* infection to ensure the timely initiation of appropriate therapy (Kuai et al., 2014; Tuon et al., 2015).

Recently, there has been increased interest in the use of obligate lytic phages as a possible alternative or supplement to traditional antibiotics for the treatment of antibiotic-resistant pathogens (Lu and Koeris, 2011). The advantages of phage therapy over currently available antibiotics include rapid self-proliferation, minimal impact on normal flora, ability to control biofilms, and low intrinsic toxicity (Kim et al., 2015). Before clinical application, potential therapeutic phages must be comprehensively examined to ensure safety and efficacy (Lin et al., 2017; Philipson et al., 2018). As yet, *E. aerogenes* bacteriophages have not been extensively investigated. Currently, there are only four reported fully-sequenced *E. aerogenes* phages: F20 (JN672684; Mishra et al., 2012), vB_EaeM_φEap-2 (NC_028695; Li et al., 2016), vB_EaeM_φEap-1 (NC_028772), and UZ1 (unclassified; Verthe et al., 2004). F20 was classified as belonging to the Siphoviridae family of T1-like viruses (Mishra et al., 2012), vB_EaeM_φEap-2 also belongs to the family Siphoviridae and is related to *Salmonella* phage FSL SP-031 (KC139518; Li et al., 2016), and vB_EaeM_φEap-1 (NC_028772) is a member of the family Podoviridae. In the current study, we focused on *E. aerogenes* phage vB_EaeM_φEap-3, a T4-like bacteriophage belonging to the genus “Kp15 virus” within the family *Myoviridae*.

## Materials and Methods

### Bacterial Host and Culture Conditions

*Enterobacter aerogenes* clinical strain 3-SP is a generous gift from Dr. Dongsheng Zhou, State Key Laboratory of Pathogen and Biosecurity, Beijing Institute of Microbiology and Epidemiology, Beijing, China, which is isolated from a human case of pneumonia at a Chinese teaching hospital (Chen et al., 2015). The isolate was originally obtained as part of routine patient care. Approval was obtained for this original procedure. Informed Oral consent was obtained, and this was sufficient for the ethics committee. Approval was not needed for this retrospective study, as approval had been obtained for the original study. Strain 3-SP was used as a host for the isolation and proliferation of phage vB_EaeM_φEap-3 and contains a pNDM-BJ01-like conjugative plasmid named p3SP-NDM that confers carbapenem resistance (Chen et al., 2015).

### Phage Isolation and Purification

Bacteriophage vB_EaeM_φEap-3 was isolated from a sewage wastewater sample from the Navy General Hospital, Beijing, China, using the double-layer overlay technique and *E. aerogenes* 3-SP as the indicator strain, as previously described (Wommack et al., 2009). Briefly, 0.22 μm filtrates of sewage samples were mixed with *E. aerogenes* 3-SP culture to enrich the phage at 37°C. The culture was centrifuged and the supernatant was filtered through a 0.22 μm pore-size membrane to remove the residual bacterial cells. Aliquots of the diluted filtrate were mixed with *E. aerogenes* culture; 3 mL of molten top soft nutrient agar (0.4% agar) was added and mixed, and overlaid on the solidified base nutrient agar (1.5% agar). Following incubation overnight at 37°C, clear phage plaques were picked from the plate. A pure phage suspension was obtained by three rounds of single-plaque purification and reinfection of the exponentially growing 3-SP strain, as reported previously (Kropinski et al., 2009b). Phage titers are expressed in plaque-forming units (PFUs)/mL and were measured using a soft agar overlay method (Kropinski et al., 2009a).

### Transmission Electron Microscopy (TEM)

The phage particle preparation was centrifuged at 20,000 ×*g* for 2 h and the resulting pellet resuspended in SM buffer (10 mM Tris-HCl, pH 7.5; 100 mM NaCl; 10 mM MgSO_4_) to a concentration of ∼10^9^ PFU/mL. Samples were processed by negative staining with 2% (wt/vol) uranyl acetate for 30 s and examined using a Tecnai Spirit 120-kV transmission electron microscope (FEI Company, Hillsboro, OR, United States) at different magnitudes to determine the phage morphology.

### Determination of Bacteriophage Host Range

The host range of phage vB_EaeM_φEap-3 was determined via the spot test method using 28 *E. aerogenes* strains, 16 enterobacterial isolates (*Enterobacter cloacae*, *n* = 2; *Enterobacter sakazakii*, *n* = 2; *Serratia marcescens*, *n* = 5; *Klebsiella pneumoniae*, *n* = 1; *Leclercia adecarboxylata*, *n* = 1; *Raoultella ornithinolytica*, *n* = 1; *Citrobacter freundii*, *n* = 1; *Shigella sonnei*, *n* = 1; *Vibrio parahaemolyticus*, *n* = 1, and *Escherichia coli*, *n* = 1), three Gram-negative non-fermenters (*Pseudomonas aeruginosa*, *n* = 1; *Acinetobacter baumannii*, *n* = 1; and *Achromobacter xylosoxidans*, *n* = 1), and one Gram-positive bacterium (*Stenotrophomonas maltophilia*, *n* = 1). Each strain was grown in 5 mL of Luria–Bertani (LB) broth at 37°C to an optical density at 600 nm (OD_600_) of 0.5 before being centrifuged at 4000 *× g* for 5 min and resuspended in 3 mL of SM buffer. A 0.2-mL aliquot of bacterial suspension was then mixed with 3 mL of molten soft-agar and poured onto an LB agar plate. After the agar had solidified, 0.01 mL of phage suspension was spotted onto the overlay. Sensitivity of the bacterium to vB_EaeM_φEap-3 infection was assessed following overnight incubation as described previously (Kutter, 2009).

### One-Step Growth Curve

The latency period and burst size of vB_EaeM_φEap-3 were determined by monitoring dynamic changes in the number of phage particles during a replicative cycle. Briefly, host strain 3-SP was grown at 37°C to mid-exponential phase (OD_600_ = 0.4–0.5) before being centrifuged at 4000 ×*g* for 10 min at 4°C. The cell pellet was then resuspended in a 0.1-volume of SM buffer. A 0.1-mL aliquot of phage suspension was then added to 0.9 mL of the bacterial suspension to achieve a multiplicity of infection of 0.01. Phages were allowed to absorb for 5 min at 37°C, and then the mixture was centrifuged twice at 16,000 ×*g* for 2 min to remove the unabsorbed phages. The mixtures were then resuspended in 10 mL of LB broth and incubated at 37°C. Samples were collected at 10-min intervals for 80 min with or without 1% chloroform and immediately diluted and plated for phage titer assays (Pajunen et al., 2000). Results are reported as the average phage titer, while the burst size was calculated by dividing the average PFU/mL of the latent period by the average PFU/mL of the last three time points of the experiment (Buttimer et al., 2018). Results are the mean of three replicates ± standard deviation.

### Influence of Physical Agents on Phage Viability

The stability of vB_EaeM_φEap-3 at different pH levels was evaluated by suspending phages at approximately 1.2 × 10^7^ PFU/mL in 1 mL of SM buffer previously adjusted with 1 M NaOH or 1 M HCl to yield a pH range from 1.0–14.0. Phage preparations were incubated at room temperature for 60 min. The stability of vB_EaeM_φEap-3 at different temperatures was determined by incubating phage preparations (∼1.2 × 10^7^ PFU/mL) at 4, 25, 37, 50, 60, 70, or 80°C for 15, 30, 45, or 60 min. After treatment, tubes were cooled and serial dilutions of each sample were tested against strain 3-SP in a double-layer agar assay to measure the lytic activity of the phage. Results are expressed as PFU/mL. Each assay was performed in triplicate and the results are the means of the three replicates.

### Extraction of Bacteriophage vB_EaeM_φEap-3 DNA

Cell debris from 500 mL of *E. aerogenes* strain 3-SP culture infected with vB_EaeM_φEap-3 was collected by low-speed centrifugation (9000 ×*g*, 10 min, 4°C). Prior to DNA extraction, DNase (1 μg/mL) and RNase (1 μg/mL) were added to the phage lysate, which was then incubated at 37°C for 30 min. Following incubation, phage particles were precipitated with 1 M NaCl and 10% (w/v) polyethylene glycol (PEG) 8000 and incubated on ice for 1 h. The mixture was then centrifuged at 9000 ×*g* for 10 min at 4°C and the pellet was resuspended in 8 mL of SM buffer. An equal volume of chloroform was added to extract the PEG and cell debris, and then centrifuged at 4000 ×*g* for 15 min. The aqueous phase containing the bacteriophage particles was recovered and transferred to a new tube and DNA was extracted with phenol-chloroform (24:1, vol/vol) and precipitated with 100% ethanol. Finally, DNA samples were dissolved in 0.5 mL of sterile ddH_2_O and stored at 4°C.

### Genome Sequencing and Bioinformatics Analysis of the Phage Genome

The genome of vB_EaeM_φEap-3 was sequenced using the Illumina HiSeq 2500 system (Illumina, United States). The reads were assembled using SSAKE (v3.8) assembly software. The final assembled sequences were searched against the protein and nucleotide databases available via the National Center for Biotechnology Information website^1^ using Basic Local Alignment Search Tool (BLAST) software (Altschul et al., 1997). BLASTP^2^ analyses were used to identify putative homologies with predicted phage proteins. Potential open reading frames (ORFs) were identified using PHASTER^3^ (Arndt et al., 2016). The annotation was numbered with reference to the “Kp15 virus” genus. Potential tRNAs were identified using tRNAscan-SE Search Server^4^ (Lowe and Chan, 2016). Computer-based predictions were checked manually. Multiple sequence alignment of the chromosomes of related bacteriophages was carried out using Mauve software^5^ (Darling et al., 2004). Phylogenetic analyses were performed using the large subunit terminase or major capsid protein sequences of bacteriophages reported by the International Committee on Taxonomy of Viruses (ICTV)^6^. Analyses were conducted using the neighbor-joining method and 1000 bootstrap replicates by ClustalW. A genome map was generated using the CLC Main Workbench, version 6.1.1 (CLC bio, Aarhus, Denmark).

## Results and Discussion

### Phage Isolation and Morphological Characterization

**FIGURE 1:**
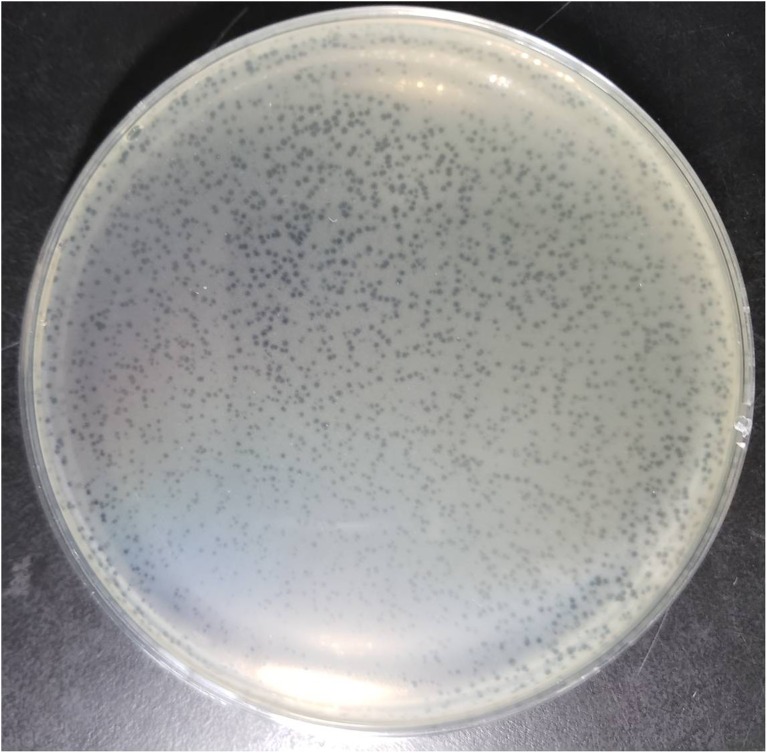
Plaques of phage vB_EaeM_φEap-3 on *Enterobacter aerogenes* strain 3-SP.

**FIGURE 2:**
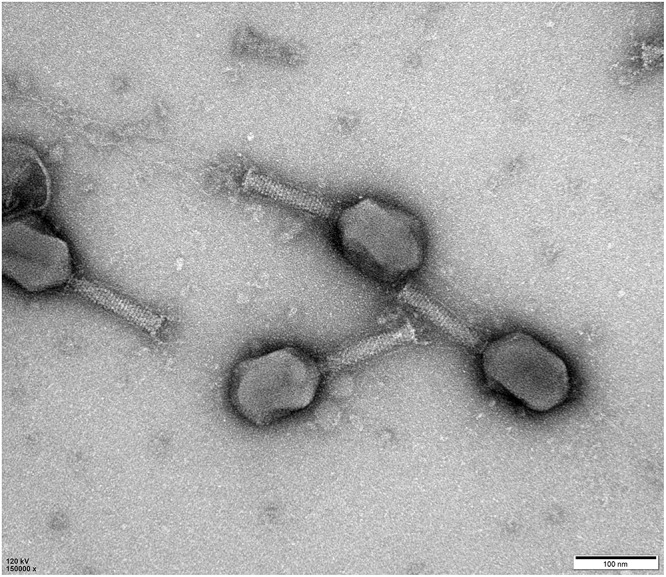
Morphology of phage vB_EaeM_φEap-3. Transmission electron micrograph of negatively stained phage vB_EaeM_φEap-3 at × 150,000 magnification. The bar indicates 100 nm.

NDM-1 carbapenemase-producing *E. aerogenes* strain 3-SP, originally isolated from a human case of pneumonia at a Chinese teaching hospital (Chen et al., 2015), was used as a host to investigate the presence of phages in a wastewater sample from the Navy General Hospital in Beijing. Double-layer overlay plates resulted in a significant number of small plaques (diameter < 1 mm) with a similar morphology, indicating the presence of a single lytic phage (Figure 1). A single plaque was selected and used for phage proliferation and purification. Using TEM, the morphology of the phage was determined. The head of the phage is prolate, with two icosahedral ends and a cylindrical mid-section measuring ∼115 nm. The head is connected by a neck with an apparent collar to a tail tube (∼110 nm long) surrounded by a contractile sheath, a baseplate, and a complex system of tail fibers and spikes (Figure 2). On the basis of morphology and according to Ackermann’s classification (Ackermann, 2009a,b), the phage was classified as belonging to the family *Myoviridae*, which comprises a quarter of tailed bacteriophages and includes the *E. coli* phage T4. The phage was named vB_EaeM_φEap-3 according to the proposed naming system, where vB = bacterial virus; Eae = abbreviation of the host species; M = myovirus; φEap-3 = name of phage (Kropinski et al., 2009b; Adriaenssens and Brister, 2017).

### Phage Host Range

A total of 48 clinical isolates (28 *E. aerogenes*, 19 non-*E. aerogenes* Gram-negative bacteria, and one Gram-positive bacterium) were used to evaluate the host range of vB_EaeM_φEap-3 (Table 1). Results demonstrated that vB_EaeM_φEap-3 had lytic activity specific to *E. aerogenes* strains (*n* = 18), with none of the other strains susceptible to infection. vB_EaeM_φEap-3 has a broader host range than the previous reported *Enterobacter* phage vB_EaeM_φEap-2 (Li et al., 2016, 2017).

**Table 1:** Host spectrum of vB_EaeM_φEap-3.

ID	ID	Lysis
*E. aerogenes*	3-SP	+
*E. aerogenes*	13208	-
*E. aerogenes*	A29864	-
*E. aerogenes*	A36179	-
*E. aerogenes*	201316724	+
*E. aerogenes*	2015-301	+
*E. aerogenes*	AH10	+
*E. aerogenes*	AH12	+
*E. aerogenes*	AH13	+
*E. aerogenes*	AH14	-
*E. aerogenes*	AH15	+
*E. aerogenes*	AH17	-
*E. aerogenes*	AH18	+
*E. aerogenes*	AH2	-
*E. aerogenes*	AH20	+
*E. aerogenes*	AH21	+
*E. aerogenes*	AH22	-
*E. aerogenes*	AH24	-
*E. aerogenes*	AH25	+
*E. aerogenes*	AH28	-
*E. aerogenes*	AH29	+
*E. aerogenes*	AH3	+
*E. aerogenes*	AH30	-
*E. aerogenes*	AH32	+
*E. aerogenes*	AH33	+
*E. aerogenes*	AH34	+
*E. aerogenes*	AH36	+
*E. aerogenes*	ATCC13048	+
*E. cloacae*	T5282	-
*E. cloacae*	TI3	-
*Cronobacter sakazakii*	45401	-
*C. sakazakii*	45402	-
*Serratia marcescens*	wk2050	-
*S. marcescens*	201315732	-
*S. marcescens*	wj-1	-
*S. marcescens*	wj-2	-
*S. marcescens*	wj-3	-
*Escherichia coli*	ATCC 25922	-
*Klebsiella pneumoniae*	ATCC BAA-1706	-
*Achromobacter xylosoxidans*	A22732	-
*Leclercia adcarboxglata*	P10164	-
*Raoultella ornithinolytica*	YNKP001	-
*Stenotrophomonas maltophilia*	9665	-
*Citrobacter freundii*	P10159	-
*Vibrio parahaemolyticus*	J5421	-
*Pseudomonas aeruginosa*	PA01	-
*Acinetobacter baumannii*	N1	-
*Shigella sonnei*	#1083	-


*+, phage-susceptible; -, phage-resistant*.

### Latency Period and Burst Size Determination

**FIGURE 3:**
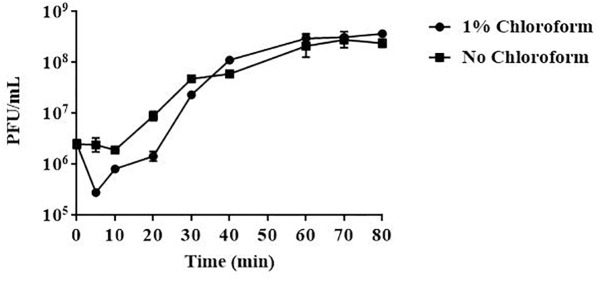
One-step growth curve of phage vB_EaeM_φEap-3. Phage vB_EaeM_φEap-3 was grown in an exponential phase culture of *Enterobacter aerogenes* strain 3-SP. Data points indicate the PFU/mL at different time points. Each data point represents the mean of three independent experiments.

Results from one-step growth experiments showed that vB_EaeM_φEap-3 was characterized by a relatively short latent period (approximately 10 min), followed by a rise period of 20 min. A growth plateau was reached within 40 min (Figure 3). The burst size of vB_EaeM_φEap-3 was calculated to be approximately 109 phage particles per infected bacterial cell.

### Sensitivity to Physical Parameters

**FIGURE 4:**
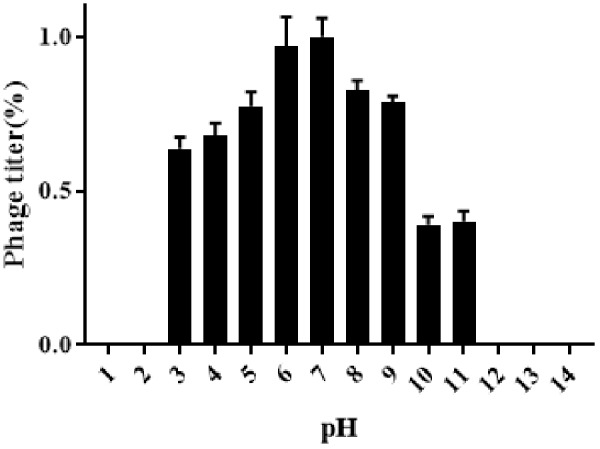
pH stability of phage vB_EaeM_φEap-3. Each data point represents the mean of three independent experiments. Standard deviations are shown as vertical lines. Results are expressed as PFU/mL.

**FIGURE 5:**
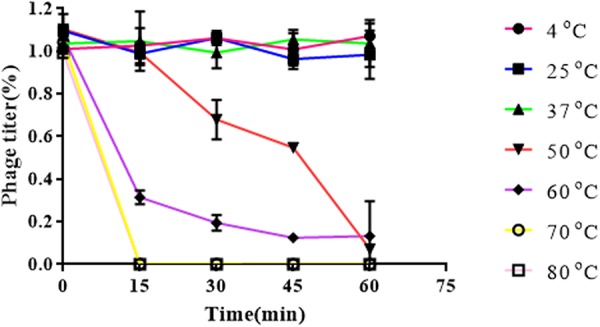
Thermal stability of phage vB_EaeM_φEap-3. Each data point represents the mean of three independent experiments. Results were expressed as PFU/mL.

Results obtained from temperature stability assays demonstrated that vB_EaeM_φEap-3 remained stable at temperatures ranging from 4–37°C. Decreases in infectivity were observed following incubation at 60 or 70°C for 15 min, while the phage was completely inactivated by incubation at 50°C for 60 min or 80°C for 15 min (Figure 4). Results of pH stability testing revealed that phage viability was mainly unaffected following incubation in buffer at pH values ranging from 6–7, while reductions of approximately 30 and 60% were detected at pH 3 and pH 11. vB_EaeM_φEap-3 was completely inactivated at pH 1–2 and pH 12–14 (Figure 5).

### Genome Analysis

**FIGURE 6:**
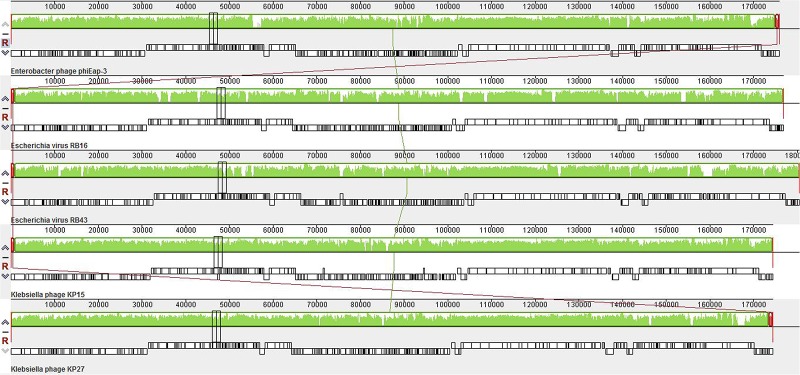
Multiple alignment of the chromosomes of phage vB_EaeM_φEap-3, coliphages RB16 and RB43, *Klebsiella* phages KP15 and KP27 obtained using Mauve software http://asap.ahabs.wisc.edu/mauve/. The height of the similarity profile corresponds to the average level of conservation in that region of the genome sequence. Completely white regions represent fragments that are not aligned or contain a particular genome-specific sequence element.

**FIGURE 7:**
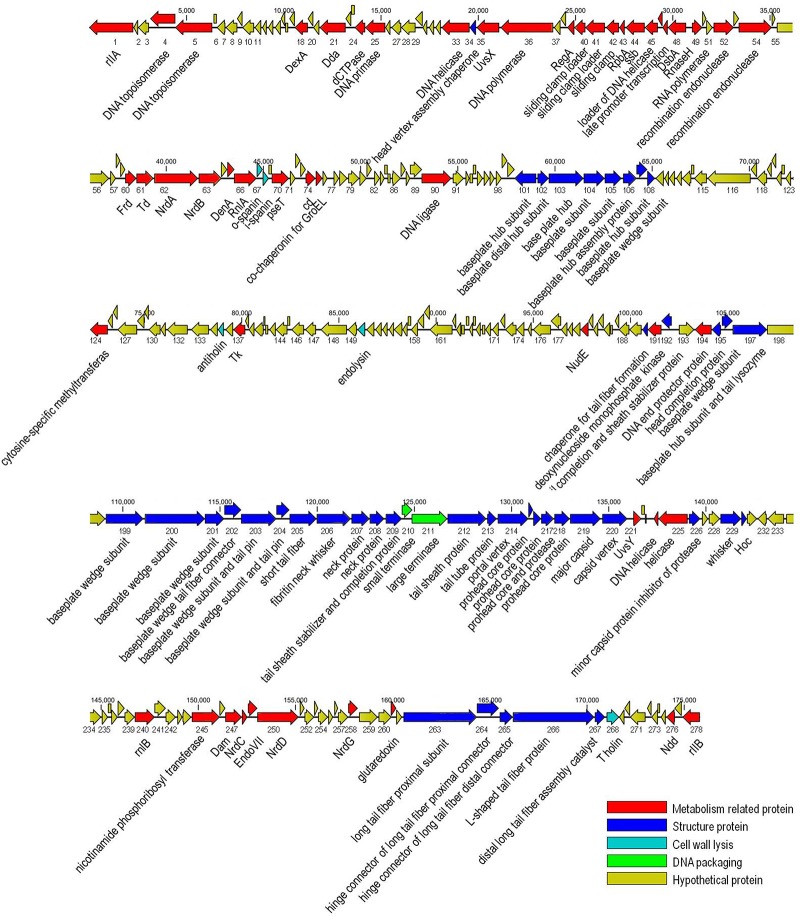
Genome map of phage vB_EaeM_φEap-3. The genome map was generated using the CLC Main Workbench, version 6.1.1 (CLC bio, Aarhus, Denmark). Predicted open reading frames are indicated by arrows, with the direction of the arrows representing the direction of transcription.

The genome of vB_EaeM_φEap-3 is composed of a double-stranded DNA molecule of 175,814 bp with a GC content of 42%. A total of 278 putative coding sequences (CDSs) were detected (Supplementary Table S1). A tRNA gene, tRNA-Met (AUG), was detected. Approximately half of the predicted CDSs (*n* = 149, 53.6%) were present on the same strand. The shortest CDS encodes a putative protein of 26 amino acid residues (*orf223*), while the longest encodes a putative protein of 1394 residues (*orf266*). A specific function (e.g., DNA metabolism, structural proteins, enzymes involved in cell lysis) could be assigned to 113 of the 278 predicted proteins (40.6%), with all sequences showing high identity to proteins from phages belonging to the “Kp15 virus” genus of the Tevenvirinae subfamily. No specific function was assigned to the remaining 165 CDSs (59.4%; Table 2). No sequences with significant similarity to known antibiotic resistance, virulence, or toxin proteins, or to elements associated with lysogeny (i.e., integrase), were identified. The presence of the *ndd* gene (*orf276*), the deduced amino acid sequence of which shared 100% identity with the nucleoid disruption protein of *Klebsiella* phage KP15, suggested a lytic lifestyle for vB_EaeM_φEap-3 (Kesik-Szeloch et al., 2013). Comparative analysis of the whole genome sequence of vB_EaeM_φEap-3 against those of phages retrieved from the NCBI databases revealed that vB_EaeM_φEap-3 is most closely related to coliphages RB16 (NC_014467; Petrov et al., 2006) and RB43 (NC_007023; Petrov et al., 2006), *Klebsiella* phages KP15 (GU295964; Kesik-Szeloch et al., 2013), KP27 (HQ918180; Kesik-Szeloch et al., 2013), Matisse (KT001918; Provasek et al., 2015), and Miro (KT001919; Mijalis et al., 2015), *Cronobacter* phage vB_CsaM_GAP161 (JN882287; Abbasifar et al., 2012), and members of the myoviral subfamily Tevenvirinae, belonging to a genus of T4-like viruses (Adriaenssens and Brister, 2017; Figure 6). A large percentage of the putative proteins that could be assigned a metabolic function were devoted to DNA metabolism and, replication (Figure 7). Seven proteins making up the basic replisome, which acts as a biological machine that can move the replication fork through model templates at *in vivo* speeds (Miller et al., 2003), were also identified in the genome of vB_EaeM_φEap-3 (Table 2). The vB_EaeM_φEap-3 genome-packaging proteins showed a high degree of similarity to those of KP15 and KP27 viruses. Phage structural proteins identified in vB_EaeM_φEap-3 genome included head proteins, whisker/neck proteins, tail proteins, baseplate proteins, and tail fiber proteins (Table 2). As a member of the “Kp15 virus” genus, the lysis system of vB_EaeM_φEap-3 is composed of four proteins (endolysin, holin, antiholin, and spanin; Table 2). vB_EaeM_φEap-3 holin (*orf268*), with one transmembrane domain, was identified as a class III holin, and belongs to the holin T superfamily group (Maciejewska et al., 2017). The lysis genes of vB_EaeM_φEap-3 show the same organization and >99% predicted amino acid sequence similarity to the same regions of phages KP15 and KP27 (Maciejewska et al., 2017).

**Table 2:** Functional categories of vB_EaeM_φEap-3 genes.

DNA metabolism	Frd (*orf60*), Td (*orf61*), NrdA (*orf62*), NrdB (*orf63*), DenA (*orf65*), cd (*orf74*), NrdC (*orf248*), NudE (*orf181*), NrdD (*orf250*), NrdG (*orf258*), Tk (*orf137*), NudE (*orf181*), NrdH (*orf261*), PseT (*orf70*), dCTPase (*orf24*), DNA methyltransferase (*orf124*), dNMP kinase (*orf191*), DNA end protector protein (*orf194*), and nicotinamide phosphoribosyl transferase (*orf245*)

DNA replication	rIIA (*orf1*), DNA topoisomerase II medium subunit (*orf4*), DNA topoisomerase II large subunit (*orf5*), DexA (*orf18*), Dda (*orf21*), DNA primase (*orf25*), DNA helicase (*orf33*), DNA polymerase (*orf36*), sliding clamp (*orf42*), loader of DNA helicase (*orf45*), DNA ligase (*orf90*), helicase (*orf224–orf225*), rIIB (*orf278*), RnlB (*orf240*), EndoVII (*orf249*), Rnh (*orf48*), and Ssb (*orf44*).
Replisome	DNA polymerase (*orf36*), sliding clamp loader (*orf40* and *orf41*), sliding clamp (*orf42*), DNA helicase (*orf33*), DNA primase (*orf25*), RnlA (*orf66*), RegA (*orf39*), and Ssb (*orf44*)
DNA maturation	The dodecameric portal protein (*orf214*), the large terminase (*orf211*), and the small terminase (*orf210*)
Head	Head completion protein (*orf195*), portal vertex protein (*orf214*), prohead core protein (*orf215*–*216*), prohead core and protease (*orf217*), prohead core protein (*orf218*), major capsid protein (*orf219*), capsid vertex protein (*orf220*), minor capsid protein inhibitor of protease (*orf226*), Hoc (*orf230*), and cochaperonin for GroEL (*orf75*)
Whisker/neck proteins	Neck protein (*orf207*–*208*), fibritin neck whiskers (*orf206*), and whisker protein (*orf229*)
Tail	Tail completion and sheath stabilizer protein (*orf192*), tail sheath stabilizer and completion protein (*orf209*), tail sheath protein (*orf212*), and tail tube protein (*orf213*)
Baseplate	The baseplate hub subunit (*orf101*, *orf107*), the baseplate distal hub subunit (*orf102*), the baseplate hub (*orf103*), the baseplate subunit (*orf104*–*105*), baseplate hub assembly protein (*orf106*), the baseplate wedge subunit (*orf108*, *orf196*, *orf199*–*201*), the baseplate hub subunit and tail lysozyme (*orf197*), baseplate wedge tail fiber connector (*orf202*), and the baseplate wedge subunit and tail pin (*orf203*–*204*)
Tail fiber	A chaperone for tail fiber formation (*orf190*), STFs (*orf205*), the long tail fiber proximal subunit (*orf263*), hinge connector of long tail fiber proximal connector (*orf264*), hinge connector of long tail fiber distal connector (*orf265*), L-shaped tail fiber protein (*orf266*), and distal long tail fiber assembly catalyst (*orf267*)
Lysis	Endolysin (*orf150*), holin (*orf268*), antiholin (*orf135*), o-spanin (*orf67*), and i-spanin (*orf68*)


### Phylogenetic Analysis

**FIGURE 8:**
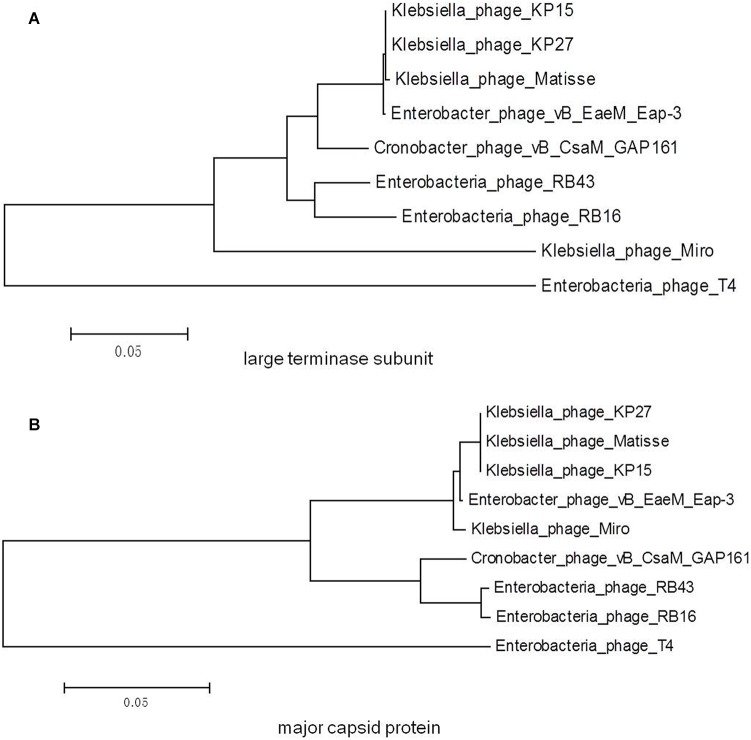
Phylogenetic trees generated based on the **(A)** large terminase subunit and **(B)** major capsid proteins of vB_EaeM_φEap-3 and homolog proteins from other members of the Tevenvirinae subfamily. Amino acid sequences were compared using ClustalW, and phylogenetic trees were generated using the neighbor-joining method.

A phylogenetic tree based on the predicted large terminase subunit amino acid sequences revealed that vB_EaeM_φEap-3 belongs to the genus “Kp15 virus” of the subfamily Tevenvirinae, family *Myoviridae* (Figure 8A). This classification was confirmed based on the analysis of the major capsid proteins (Figure 8B).

## Conclusion

The spread of antibiotic-resistance among bacterial pathogens poses a serious problem in a clinical setting because of the lack of available treatment options. In particular, carbapenem resistance and its growing association with a multidrug-resistant phenotype in Enterobacteriaceae, including *E. aerogenes*, has become a major clinical challenge. As an important opportunistic pathogen, *E. aerogenes* can cause nosocomial outbreaks and invasive infections such as septicemia (Kuai et al., 2014). In this work, we describe a lytic bacteriophage characterized by its specific lytic activity toward *E. aerogenes*. Morphological characterization performed by TEM showed that vB_EaeM_φEap-3 is a member of the family *Myoviridae*, while phylogenetic analysis using previously verified markers (Ackermann et al., 2011; Cheepudom et al., 2015) suggested that it belongs to the novel genus “Kp15 virus” of the Tevenvirinae subfamily. Physiological characterization showed that vB_EaeM_φEap-3 is characterized by a relatively short latent period and a burst size of 109 phage particles per infected bacterial cell. Results of temperature and pH stability testing also expand our knowledge of this novel phage. These features, together with the host specificity, the close genetic relatedness to the strictly lytic genus “Kp15 virus” phages, and the absence of genes associated with lysogeny, make vB_EaeM_φEap-3 an excellent candidate for potential clinical applications, such as decontamination or therapy. Finally, the results confirmed that vB_EaeM_φEap-3 is a promising candidate to hinder the colonization of *E. aerogenes*.

## Nucleotide Sequence Accession Number

The complete genome sequence of phage vB_EaeM_φEap-3 is available in GenBank under accession number KT321315.

## Author Contributions

JZ and ZZ did the experiments and contributed equally to this study as joint first authors. CT, XC, LH, XW, HL, WL, and AJ analyzed the data. RF, ZY, and JY provided the bacterial strains. XZ managed the project and designed the experiments. JZ and XZ wrote the article.

## Conflict of Interest Statement

The authors declare that the research was conducted in the absence of any commercial or financial relationships that could be construed as a potential conflict of interest.

## Footnotes

1 http://www.ncbi.nlm.nih.gov/

2 http://www.ncbi.nlm.nih.gov/BLAST/

3 http://phaster.ca

4 http://lowelab.ucsc.edu/tRNAscan-SE/

5 http://asap.ahabs.wisc.edu/mauve/

6 https://talk.ictvonline.org/taxonomy/

## References

[B1] Abbasifar R. Kropinski A. M. Sabour P. M. Ackermann H. W. Lingohr E. J. Griffiths M. W. (2012). Complete genome sequence of *Cronobacter sakazakii* bacteriophage vB_CsaM_GAP161. *J. Virol.* 86 13806–13807. 10.1128/JVI.02546-12 23166229 PMC3503131

[B2] Ackermann H. W. (2009a). Basic phage electron microscopy. *Methods Mol. Biol.* 501 113–126. 19066816 10.1007/978-1-60327-164-6_12

[B3] Ackermann H. W. (2009b). Phage Classification and Characterization. *Methods Mol. Biol.* 501 127–140. 19066817 10.1007/978-1-60327-164-6_13

[B4] Ackermann H. W. Krisch H. M. Comeau A. M. (2011). Morphology and genome sequence of phage varphi1402: a dwarf myovirus of the predatory bacterium *Bdellovibrio* bacteriovorus. *Bacteriophage* 1 138–142. 22164347 10.4161/bact.1.3.15769 PMC3225778

[B5] Adriaenssens E. Brister J. R. (2017). How to name and classify your phage: an informal guide. *Viruses* 9:E70. 10.3390/v9040070 28368359 PMC5408676

[B6] Altschul S. F. Madden T. L. Schaffer A. A. Zhang J. Zhang Z. Miller W. (1997). Gapped BLAST and PSI-BLAST: a new generation of protein database search programs. *Nucleic Acids Res.* 25 3389–3402. 9254694 10.1093/nar/25.17.3389 PMC146917

[B7] Arndt D. Grant J. R. Marcu A. Sajed T. Pon A. Liang Y. (2016). PHASTER: a better, faster version of the PHAST phage search tool. *Nucleic Acids Res.* 44 W16–W21. 10.1093/nar/gkw387 27141966 PMC4987931

[B8] Buttimer C. Lucid A. Neve H. Franz C. O’mahony J. Turner D. (2018). *Pectobacterium atrosepticum* Phage vB_PatP_CB5: a Member of the Proposed Genus ’Phimunavirus’. *Viruses* 10:E394. 10.3390/v10080394 30050020 PMC6115819

[B9] Cheepudom J. Lee C. C. Cai B. Meng M. (2015). Isolation, characterization, and complete genome analysis of P1312, a thermostable bacteriophage that infects Thermobifida fusca. *Front. Microbiol.* 6:959. 10.3389/fmicb.2015.00959 26441893 PMC4569894

[B10] Chen Z. Li H. Feng J. Li Y. Chen X. Guo X. (2015). NDM-1 encoded by a pNDM-BJ01-like plasmid p3SP-NDM in clinical *Enterobacter aerogenes*. *Front. Microbiol.* 6:294. 10.3389/fmicb.2015.00294 25926823 PMC4396501

[B11] Darling A. C. Mau B. Blattner F. R. Perna N. T. (2004). Mauve: multiple alignment of conserved genomic sequence with rearrangements. *Genome Res.* 14 1394–1403. 15231754 10.1101/gr.2289704 PMC442156

[B12] Davin-Regli A. Pages J. M. (2015). *Enterobacter aerogenes* and *Enterobacter cloacae*; versatile bacterial pathogens confronting antibiotic treatment. *Front. Microbiol.* 6:392. 10.3389/fmicb.2015.00392 26042091 PMC4435039

[B13] Kesik-Szeloch A. Drulis-Kawa Z. Weber-Dabrowska B. Kassner J. Majkowska-Skrobek G. Augustyniak D. (2013). Characterising the biology of novel lytic bacteriophages infecting multidrug resistant *Klebsiella pneumoniae*. *Virol. J.* 10:100. 10.1186/1743-422X-10-100 23537199 PMC3620542

[B14] Kim M. S. Kim Y. D. Hong S. S. Park K. Ko K. S. Myung H. (2015). Phage-encoded colanic acid-degrading enzyme permits lytic phage infection of a capsule-forming resistant mutant *Escherichia coli* strain. *Appl. Environ. Microbiol.* 81 900–909. 10.1128/AEM.02606-14 25416767 PMC4292498

[B15] Kropinski A. M. Mazzocco A. Waddell T. E. Lingohr E. Johnson R. P. (2009a). Enumeration of bacteriophages by double agar overlay plaque assay. *Methods Mol. Biol.* 501 69–76. 10.1007/978-1-60327-164-6_7 19066811

[B16] Kropinski A. M. Prangishvili D. Lavigne R. (2009b). Position paper: the creation of a rational scheme for the nomenclature of viruses of Bacteria and Archaea. *Environ. Microbiol.* 11 2775–2777. 19519870 10.1111/j.1462-2920.2009.01970.x

[B17] Kuai S. Shao H. Huang L. Pei H. Lu Z. Wang W. (2014). KPC-2 carbapenemase and DHA-1 AmpC determinants carried on the same plasmid in *Enterobacter aerogenes*. *J. Med. Microbiol.* 63 367–370. 10.1099/jmm.0.054627-0 24173427

[B18] Kutter E. (2009). Phage host range and efficiency of plating. *Methods Mol. Biol.* 501 141–149. 19066818 10.1007/978-1-60327-164-6_14

[B19] Li E. Wei X. Ma Y. Yin Z. Li H. Lin W. (2016). Isolation and characterization of a bacteriophage phiEap-2 infecting multidrug resistant *Enterobacter aerogenes*. *Sci. Rep.* 6:28338. 10.1038/srep28338 27320081 PMC4913238

[B20] Li E. Wei X. Ma Y. Yin Z. Li H. Lin W. (2017). Corrigendum: isolation and characterization of a bacteriophage phiEap-2 infecting multidrug resistant *Enterobacter aerogenes*. *Sci. Rep.* 7:46805. 28485399 10.1038/srep46805 PMC5422924

[B21] Lin D. M. Koskella B. Lin H. C. (2017). Phage therapy: an alternative to antibiotics in the age of multi-drug resistance. *World J. Gastrointest Pharmacol. Ther.* 8 162–173. 10.4292/wjgpt.v8.i3.162 28828194 PMC5547374

[B22] Lowe T. M. Chan P. P. (2016). tRNAscan-SE On-line: integrating search and context for analysis of transfer RNA genes. *Nucleic Acids Res.* 44 W54–W57. 10.1093/nar/gkw413 27174935 PMC4987944

[B23] Lu T. K. Koeris M. S. (2011). The next generation of bacteriophage therapy. *Curr. Opin. Microbiol.* 14 524–531. 10.1016/j.mib.2011.07.028 21868281

[B24] Maciejewska B. Roszniowski B. Espaillat A. Kesik-Szeloch A. Majkowska-Skrobek G. Kropinski A. M. (2017). *Klebsiella* phages representing a novel clade of viruses with an unknown DNA modification and biotechnologically interesting enzymes. *Appl. Microbiol. Biotechnol.* 101 673–684. 10.1007/s00253-016-7928-3 27766357 PMC5219037

[B25] Mijalis E. M. Lessor L. E. Cahill J. L. Rasche E. S. Kuty Everett G. F. (2015). Complete Genome Sequence of Klebsiella pneumoniae Carbapenemase-Producing K. pneumoniae Myophage Miro. *Genome Announc.* 3:e994-15. 10.1128/genomeA.01137-15 26430050 PMC4591322

[B26] Miller E. S. Kutter E. Mosig G. Arisaka F. Kunisawa T. Ruger W. (2003). Bacteriophage T4 genome. *Microbiol. Mol. Biol. Rev.* 67 86–156. 12626685 10.1128/MMBR.67.1.86-156.2003 PMC150520

[B27] Mishra C. K. Choi T. J. Kang S. C. (2012). Isolation and characterization of a bacteriophage F20 virulent to *Enterobacter aerogenes*. *J. Gen. Virol.* 93 2310–2314. 10.1099/vir.0.043562-0 22764320

[B28] Pajunen M. Kiljunen S. Skurnik M. (2000). Bacteriophage phiYeO3-12, specific for *Yersinia enterocolitica* serotype O:3, is related to coliphages T3 and T7. *J. Bacteriol.* 182 5114–5120. 10960095 10.1128/jb.182.18.5114-5120.2000 PMC94659

[B29] Petrov V. M. Nolan J. M. Bertrand C. Levy D. Desplats C. Krisch H. M. (2006). Plasticity of the gene functions for DNA replication in the T4-like phages. *J. Mol. Biol.* 361 46–68. 16828113 10.1016/j.jmb.2006.05.071

[B30] Philipson C. W. Voegtly L. J. Lueder M. R. Long K. A. Rice G. K. Frey K. G. (2018). Characterizing phage genomes for therapeutic applications. *Viruses* 10:E188. 10.3390/v10040188 29642590 PMC5923482

[B31] Provasek V. E. Lessor L. E. Cahill J. L. Rasche E. S. Kuty Everett G. F. (2015). Complete genome sequence of carbapenemase-producing *Klebsiella pneumoniae* myophage matisse. *Genome Announc.* 3 e1136–15. 10.1128/genomeA.01136-15 26430049 PMC4591321

[B32] Qin X. Yang Y. Hu F. Zhu D. (2014). Hospital clonal dissemination of *Enterobacter aerogenes* producing carbapenemase KPC-2 in a Chinese teaching hospital. *J. Med. Microbiol.* 63 222–228. 10.1099/jmm.0.064865-0 24273320

[B33] Tuon F. F. Scharf C. Rocha J. L. Cieslinsk J. Becker G. N. Arend L. N. (2015). KPC-producing *Enterobacter aerogenes* infection. *Braz. J. Infect. Dis.* 19 324–327. 10.1016/j.bjid.2015.01.003 25722130 PMC9425374

[B34] Verthe K. Possemiers S. Boon N. Vaneechoutte M. Verstraete W. (2004). Stability and activity of an *Enterobacter aerogenes*-specific bacteriophage under simulated gastro-intestinal conditions. *Appl. Microbiol. Biotechnol.* 65 465–472. 10.1007/s00253-004-1585-7 14991251

[B35] Wommack K. E. Williamson K. E. Helton R. R. Bench S. R. Winget D. M. (2009). Methods for the isolation of viruses from environmental samples. *Methods Mol. Biol.* 501 3–14. 10.1007/978-1-60327-164-6_1 19066805

