# Supplementary material for: Characterizing the Biology of Lytic Bacteriophage vB_EaeM_φEap-3 Infecting Multidrug-Resistant Enterobacter aerogenes
Source: Front Microbiol. 2019 Mar 5;10:420. doi: 10.3389/fmicb.2019.00420 (PMC6412083; doi:10.3389/fmicb.2019.00420)
Supplement: Supplementary file 1 [file Table_1.DOCX]

Supplementary Table 1. vB_EaeM_φEap-3 gene annotations

| ORFs | amino acid length | % identity | E-value | Score | Annotation |
| --- | --- | --- | --- | --- | --- |
| ORF1 | 753 | 98.8 | 0 | 1546 | rIIA, protector from prophage-induced early lysis (KP27) |
| ORF2 | 78 | 89.74 | 2E-42 | 140 | Unknown (KP27) |
| ORF3 | 184 | 98.91 | 1E-133 | 379 | Unknown (KP27) |
| ORF4 | 428 | 99.53 | 0 | 881 | DNA topoisomerase II medium subunit (KP27) |
| ORF5 | 632 | 99.84 | 0 | 1306 | DNA topoisomerase II large subunit (KP15) |
| ORF6 | 54 | 100 | 2E-32 | 113 | Unknown (KP27) |
| ORF7 | 146 | 98.63 | 3E-106 | 306 | Unknown (KP15) |
| ORF8 | 197 | 87.82 | 1E-124 | 357 | Unknown (KP27) |
| ORF9 | 92 | 100 | 5E-64 | 196 | Unknown (KP15) |
| ORF10 | 198 | 98.99 | 4E-146 | 412 | Unknown (KP15) |
| ORF11 | 109 | 98.17 | 1E-74 | 223 | Unknown (KP27) |
| ORF12 | 74 | 98.65 | 2E-46 | 150 | Unknown (KP27) |
| ORF13 | 87 | 97.7 | 7E-56 | 174 | Unknown (KP15) |
| ORF14 | 99 | 97.98 | 1E-64 | 197 | Unknown (KP27) |
| ORF15 | 74 | 93.24 | 3E-45 | 146 | Unknown (KP27) |
| ORF16 | 49 | 100 | 1E-26 | 97.8 | Unknown (KP15) |
| ORF17 | 93 | 96.77 | 4E-59 | 183 | Unknown (KP15) |
| ORF18 | 223 | 100 | 1E-165 | 463 | DexA, exonuclease A (KP15) |
| ORF19 | 94 | 100 | 3E-64 | 196 | Unknown (KP27) |
| ORF20 | 102 | 100 | 4E-68 | 207 | Unknown (KP15) |
| ORF21 | 442 | 100 | 0 | 922 | Dda, DNA helicase (KP27) |
| ORF22 | 96 | 98.96 | 8E-63 | 192 | Unknown (KP15) |
| ORF23 | 61 | 98.28 | 3E-32 | 113 | UnknownA (KP27) |
| ORF24 | 178 | 100 | 2E-131 | 372 | dCTPase (KP15) |
| ORF25 | 341 | 99.71 | 0 | 707 | DNA primase subunit (KP15) |
| ORF26 | 86 | 100 | 5E-58 | 180 | Unknown (KP27) |
| ORF27 | 165 | 97.53 | 2E-114 | 328 | Unknown (KP15) |
| ORF28 | 243 | 100 | 0 | 511 | Unknown (KP15) |
| ORF29 | 129 | 96.12 | 2E-89 | 263 | Unknown (KP15) |
| ORF30 | 78 | 100 | 2E-46 | 150 | Unknown (KP15) |
| ORF31 | 84 | 100 | 1E-53 | 169 | Unknown (KP15) |
| ORF32 | 89 | 96.63 | 2E-46 | 150 | Unknown (KP15) |
| ORF33 | 478 | 99.79 | 0 | 993 | DNA helicase (KP15) |
| ORF34 | 106 | 100 | 4E-71 | 214 | head vertex assembly chaperone (KP15) |
| ORF35 | 385 | 99.48 | 0 | 791 | UvsX, RecA-like recombination protein (KP15) |
| ORF36 | 899 | 99.67 | 0 | 1872 | DNA polymerase (KP15) |
| ORF37 | 109 | 100 | 8E-74 | 221 | Unknown (KP15) |
| ORF38 | 95 | 97.89 | 4E-63 | 193 | Unknown (KP15) |
| ORF39 | 120 | 100 | 2E-83 | 246 | RegA (KP27) |
| ORF40 | 187 | 99.47 | 7E-133 | 377 | sliding clamp loader (KP15) |
| ORF41 | 332 | 100 | 0 | 683 | sliding clamp loader (KP15) |
| ORF42 | 221 | 100 | 2E-156 | 440 | sliding clamp (KP15) |
| ORF43 | 89 | 100 | 5E-58 | 180 | RpbA, RNA polymerase binding protein (KP15) |
| ORF44 | 323 | 99.69 | 0 | 656 | ssb, ssDNA binding protein (KP27) |
| ORF45 | 219 | 99.54 | 5E-155 | 436 | loader of DNA helicase (KP27) |
| ORF46 | 81 | 100 | 5E-53 | 167 | late promoter transcription (KP15) |
| ORF47 | 87 | 98.85 | 2E-55 | 173 | DsbA, dsDNA binding (KP15) |
| ORF48 | 311 | 100 | 0 | 640 | Rnase H (KP15) |
| ORF49 | 175 | 100 | 6E-129 | 366 | RNA polymerase sigma factor (KP15) |
| ORF50 | 71 | 100 | 3E-42 | 139 | Unknown (KP15) |
| ORF51 | 108 | 100 | 4E-73 | 219 | Unknown (KP15) |
| ORF52 | 343 | 99.42 | 0 | 714 | recombination endonuclease subunit (KP27) |
| ORF53 | 95 | 100 | 4E-63 | 193 | Unknown (KP15) |
| ORF54 | 565 | 97.52 | 0 | 1135 | recombination endonuclease subunit (KP27) |
| ORF55 | 63 | 96.83 | 1E-36 | 124 | Unknown (KP15) |
| ORF56 | 569 | 99.82 | 0 | 1177 | Unknown (KP27) |
| ORF57 | 96 | 100 | 1E-65 | 200 | Unknown (KP27) |
| ORF58 | 76 | 98.68 | 9E-48 | 153 | Unknown (KP15) |
| ORF59 | 85 | 100 | 2E-56 | 176 | Unknown (KP15) |
| ORF60 | 192 | 94.79 | 2E-133 | 379 | Frd, dihydrofolate reductase (KP15) |
| ORF61 | 291 | 99.66 | 0 | 604 | Td, thymidylate synthetase (KP15) |
| ORF62 | 750 | 100 | 0 | 1568 | NrdA, ribonucleotide reductase A subunit (KP15) |
| ORF63 | 391 | 99.74 | 0 | 823 | NrdB, aerobic ribonucleotide reductase B subunit (KP27) |
| ORF64 | 106 | 100 | 6E-75 | 224 | Unknown (KP15) |
| ORF65 | 121 | 100 | 8E-86 | 253 | DenA, endonuclease II (KP15) |
| ORF66 | 385 | 99.22 | 0 | 785 | RnlA, RNA ligase 1 (KP15) |
| ORF67 | 103 | 100 | 2E-68 | 207 | o-spanin (KP15) |
| ORF68 | 104 | 99.04 | 3E-69 | 209 | i-spanin (KP15) |
| ORF69 | 57 | 98.25 | 3E-32 | 112 | Unknown (KP27) |
| ORF70 | 294 | 99.66 | 0 | 615 | pseT, 3' phosphatase, 5' polynucleotide kinase (KP27) |
| ORF71 | 95 | 96.84 | 8E-62 | 190 | Unknown (KP27) |
| ORF72 | 88 | 98.86 | 1E-56 | 176 | Unknown (KP15) |
| ORF73 | 94 | 100 | 2E-64 | 196 | Unknown (KP27) |
| ORF74 | 168 | 100 | 3E-125 | 356 | cd, dCMP deaminase (KP27) |
| ORF75 | 113 | 100 | 1E-76 | 229 | co-chaperonin for GroEL, Head assembly (KP27) |
| ORF76 | 83 | 100 | 6E-54 | 169 | Unknown (KP15) |
| ORF77 | 93 | 95.7 | 1E-60 | 187 | Unknown (KP15) |
| ORF78 | 140 | 97.14 | 7E-95 | 277 | Unknown (KP15) |
| ORF79 | 188 | 98.94 | 2E-136 | 386 | Unknown (KP27) |
| ORF80 | 120 | 100 | 8E-83 | 245 | Unknown (KP27) |
| ORF81 | 89 | 97.75 | 4E-57 | 178 | Unknown (KP27) |
| ORF82 | 97 | 98.97 | 7E-64 | 196 | Unknown (KP15) |
| ORF83 | 79 | 100 | 4E-53 | 167 | Unknown (KP15) |
| ORF84 | 56 | 98.21 | 5E-34 | 117 | Unknown (KP15) |
| ORF85 | 50 | 100 | 7E-28 | 101 | Unknown (KP15) |
| ORF86 | 146 | 100 | 4E-107 | 308 | Unknown (KP15) |
| ORF87 | 87 | 98.85 | 7E-56 | 174 | Unknown (KP15) |
| ORF88 | 73 | 100 | 1E-46 | 150 | Unknown (KP15) |
| ORF89 | 204 | 100 | 4E-152 | 427 | Unknown (KP27) |
| ORF90 | 509 | 100 | 0 | 1035 | DNA ligase (KP15) |
| ORF91 | 189 | 97.88 | 8E-137 | 387 | Unknown (KP15) |
| ORF92 | 67 |  |  |  | Unknown |
| ORF93 | 58 |  |  |  | Unknown |
| ORF94 | 54 |  |  |  | Unknown |
| ORF95 | 100 |  |  |  | Unknown |
| ORF96 | 96 | 51.09 | 2E-21 | 88.2 | Unknown (KP15) |
| ORF97 | 84 | 46.43 | 3E-15 | 71.6 | Unknown (T1) |
| ORF98 | 99 |  |  |  | Unknown |
| ORF99 | 97 | 100 | 4E-56 | 176 | Unknown (KP15) |
| ORF100 | 125 | 96.8 | 1E-85 | 253 | Unknown (KP15) |
| ORF101 | 369 | 99.46 | 0 | 764 | baseplate hub subunit (KP27) |
| ORF102 | 191 | 98.95 | 2E-136 | 387 | baseplate distal hub subunit (KP15) |
| ORF103 | 595 | 94.12 | 0 | 1101 | base plate hub (KP27) |
| ORF104 | 357 | 99.16 | 0 | 726 | baseplate subunit (KP15) |
| ORF105 | 287 | 100 | 0 | 590 | baseplate subunit (KP15) |
| ORF106 | 230 | 100 | 1E-166 | 467 | baseplate hub assembly protein (KP15) |
| ORF107 | 184 | 100 | 3E-135 | 383 | baseplate hub subunit (KP27) |
| ORF108 | 130 | 99.23 | 1E-89 | 263 | baseplate wedge subunit (KP15) |
| ORF109 | 142 | 98.59 | 2E-100 | 292 | Unknown (KP15) |
| ORF110 | 74 | 100 | 5E-48 | 154 | Unknown (KP15) |
| ORF111 | 98 | 42.22 | 1E-17 | 79.3 | Unknown (KP15) |
| ORF112 | 94 | 100 | 1E-62 | 192 | Unknown (KP15) |
| ORF113 | 156 | 95.51 | 9E-90 | 266 | Unknown (KP15) |
| ORF114 | 52 | 98.08 | 2E-28 | 102 | Unknown (KP15) |
| ORF115 | 216 | 99.07 | 3E-156 | 439 | Unknown (KP27) |
| ORF116 | 738 | 100 | 0 | 1517 | Unknown (KP15) |
| ORF117 | 106 | 99.06 | 5E-71 | 214 | Unknown (KP15) |
| ORF118 | 171 | 98.25 | 5E-126 | 358 | Unknown (KP15) |
| ORF119 | 80 | 87.34 | 2E-45 | 148 | Unknown (KP27) |
| ORF120 | 79 | 93.59 | 3E-47 | 152 | Unknown (vB_CsaM_GAP161) |
| ORF121 | 94 | 95.74 | 1E-61 | 189 | Unknown (KP15) |
| ORF122 | 55 | 98.18 | 3E-29 | 105 | Unknown (KP15) |
| ORF123 | 92 | 100 | 2E-63 | 194 | Unknown (vB_CsaM_GAP161) |
| ORF124 | 303 | 99.34 | 0 | 627 | cytosine-specific methyltransferase (KP27) |
| ORF125 | 86 | 90.7 | 3E-48 | 155 | Unknown (KP27) |
| ORF126 | 81 | 100 | 5E-54 | 169 | Unknown (KP15) |
| ORF127 | 335 | 98.81 | 0 | 691 | Unknown (KP15) |
| ORF128 | 125 | 100 | 1E-88 | 260 | Unknown (KP15) |
| ORF129 | 75 | 98.67 | 7E-48 | 153 | Unknown (KP15) |
| ORF130 | 201 | 97.51 | 4E-140 | 397 | Unknown (KP15) |
| ORF131 | 89 | 92.13 | 2E-57 | 179 | Unknown (KP15) |
| ORF132 | 352 | 96.31 | 0 | 692 | Unknown (KP27) |
| ORF133 | 304 | 100 | 0 | 623 | Unknown (KP15) |
| ORF134 | 127 | 100 | 1E-89 | 263 | Unknown (KP15) |
| ORF135 | 100 | 98 | 3E-69 | 209 | Antiholin (KP15) |
| ORF136 | 130 | 100 | 2E-92 | 270 | Unknown (KP27) |
| ORF137 | 202 | 98.51 | 3E-148 | 417 | Tk, thymidine kinase (KP15) |
| ORF138 | 65 | 96.92 | 3E-41 | 136 | Unknown (KP27) |
| ORF139 | 95 | 95.79 | 2E-60 | 186 | Unknown (KP15) |
| ORF140 | 157 | 100 | 4E-112 | 322 | Unknown (KP27) |
| ORF141 | 32 |  |  |  | Unknown |
| ORF142 | 48 | 76.47 | 3E-18 | 77 | Unknown (KP27) |
| ORF143 | 107 | 100 | 7E-74 | 221 | Unknown (KP15) |
| ORF144 | 196 | 98.98 | 4E-145 | 409 | Unknown (KP15) |
| ORF145 | 48 | 100 | 4E-28 | 102 | Unknown (KP15) |
| ORF146 | 213 | 99.53 | 4E-156 | 438 | Vs.1 (KP15) |
| ORF147 | 184 | 98.37 | 6E-132 | 374 | Unknown (KP15) |
| ORF148 | 447 | 99.55 | 0 | 922 | Unknown (KP15) |
| ORF149 | 152 | 99.34 | 4E-111 | 319 | Unknown (KP15) |
| ORF150 | 131 | 100 | 7E-91 | 266 | endolysin (KP27) |
| ORF151 | 133 | 87.97 | 1E-84 | 251 | Unknown (KP27) |
| ORF152 | 107 | 98.13 | 4E-72 | 217 | Unknown (KP27) |
| ORF153 | 87 | 98.85 | 8E-58 | 179 | Unknown (KP27) |
| ORF154 | 86 | 98.84 | 3E-56 | 176 | Unknown (KP27) |
| ORF155 | 143 | 100 | 5E-104 | 300 | Unknown (KP27) |
| ORF156 | 84 | 56.25 | 2E-26 | 100 | Unknown (Pseudomonas aeruginosa) |
| ORF157 | 57 | 98.25 | 9E-34 | 116 | Unknown (KP27) |
| ORF158 | 115 | 95.61 | 5E-75 | 225 | Unknown (KP27) |
| ORF159 | 113 | 48.57 | 3E-30 | 112 | Unknown (vB_CsaM_GAP161) |
| ORF160 | 98 | 48.42 | 1E-22 | 92 | Unknown (KP27) |
| ORF161 | 389 | 97.69 | 0 | 764 | Unknown (KP15) |
| ORF162 | 67 | 86.57 | 5E-37 | 125 | Unknown (KP27) |
| ORF163 | 85 | 96.47 | 2E-53 | 168 | Unknown (KP15) |
| ORF164 | 57 |  |  |  | Unknown |
| ORF165 | 56 | 83.64 | 1E-27 | 100 | Unknown (RB43) |
| ORF166 | 65 | 100 | 1E-40 | 134 | Unknown (KP15) |
| ORF167 | 49 | 77.55 | 2E-22 | 87.8 | Unknown (RB43) |
| ORF168 | 83 | 91.57 | 1E-49 | 159 | Unknown (KP15) |
| ORF169 | 53 | 79.25 | 4E-23 | 89.7 | Unknown (RB43) |
| ORF170 | 92 | 97.83 | 1E-60 | 187 | Unknown (KP15) |
| ORF171 | 110 |  |  |  | Unknown |
| ORF172 | 86 | 94.19 | 3E-54 | 170 | Unknown (KP27) |
| ORF173 | 191 | 97.91 | 1E-138 | 392 | Unknown (KP15) |
| ORF174 | 132 | 98.48 | 6E-93 | 273 | Unknown (KP15) |
| ORF175 | 66 | 100 | 2E-42 | 139 | Unknown (KP27) |
| ORF176 | 304 | 97.37 | 0 | 626 | Unknown (KP27) |
| ORF177 | 175 | 98.86 | 1E-128 | 365 | Unknown (KP15) |
| ORF178 | 107 | 98.13 | 8E-74 | 221 | Unknown (KP15) |
| ORF179 | 85 | 100 | 2E-56 | 176 | Unknown (KP15) |
| ORF180 | 127 | 99.21 | 3E-89 | 262 | Unknown (KP15) |
| ORF181 | 135 | 98.52 | 1E-96 | 281 | NudE, nudix hydrolase (KP15) |
| ORF182 | 81 | 88.89 | 2E-45 | 147 | Unknown (KP27) |
| ORF183 | 83 | 97.59 | 4E-55 | 172 | Unknown (KP27) |
| ORF184 | 97 | 100 | 1E-67 | 205 | Unknown (KP15) |
| ORF185 | 111 | 93.69 | 2E-73 | 221 | Unknown (KP27) |
| ORF186 | 42 | 78.57 | 2E-15 | 69.7 | Unknown (KP15) |
| ORF187 | 74 | 100 | 3E-27 | 100 | Unknown (KP15) |
| ORF188 | 179 | 99.44 | 3E-131 | 372 | Unknown (KP27) |
| ORF189 | 207 | 99.03 | 2E-149 | 421 | Unknown (KP27) |
| ORF190 | 90 | 97.67 | 1E-51 | 164 | chaperone for tail fiber formation (KP27) |
| ORF191 | 231 | 96.1 | 3E-166 | 465 | deoxynucleoside monophosphate kinase (KP27) |
| ORF192 | 176 | 99.43 | 7E-129 | 366 | tail completion and sheath stabilizer protein (KP15) |
| ORF193 | 262 | 100 | 0 | 533 | Unknown (KP27) |
| ORF194 | 284 | 100 | 0 | 581 | DNA end protector protein (KP27) |
| ORF195 | 155 | 100 | 8E-113 | 324 | head completion protein (KP15) |
| ORF196 | 184 | 99.46 | 2E-134 | 381 | baseplate wedge subunit (KP27) |
| ORF197 | 589 | 99.32 | 0 | 1201 | baseplate hub subunit and tail lysozyme (KP27) |
| ORF198 | 688 | 99.56 | 0 | 1424 | Unknown (KP15) |
| ORF199 | 641 | 99.53 | 0 | 1306 | baseplate wedge subunit (KP27) |
| ORF200 | 1028 | 99.22 | 0 | 2104 | baseplate wedge subunit (KP15) |
| ORF201 | 330 | 99.7 | 0 | 679 | baseplate wedge subunit (KP15) |
| ORF202 | 287 | 100 | 0 | 586 | baseplate wedge tail fiber connector (KP27) |
| ORF203 | 605 | 99.17 | 0 | 1237 | baseplate wedge subunit and tail pin (KP27) |
| ORF204 | 222 | 99.55 | 2E-162 | 454 | baseplate wedge subunit and tail pin (KP15) |
| ORF205 | 461 | 97.61 | 0 | 927 | short tail fibers (KP27) |
| ORF206 | 583 | 98.63 | 0 | 1155 | fibritin neck whiskers (KP27) |
| ORF207 | 308 | 99.03 | 0 | 627 | neck protein (KP15) |
| ORF208 | 248 | 100 | 0 | 509 | neck protein (KP15) |
| ORF209 | 274 | 98.18 | 0 | 565 | tail sheath stabilizer and completion protein (KP15) |
| ORF210 | 179 | 100 | 8E-129 | 366 | small terminase protein (KP15) |
| ORF211 | 609 | 99.84 | 0 | 1274 | large terminase protein (KP15) |
| ORF212 | 663 | 98.64 | 0 | 1347 | tail sheath protein (KP15) |
| ORF213 | 161 | 100 | 2E-117 | 336 | tail tube protein (KP15) |
| ORF214 | 524 | 100 | 0 | 1084 | portal vertex protein (KP15) |
| ORF215 | 80 | 100 | 7E-47 | 151 | prohead core protein (KP15) |
| ORF216 | 134 | 100 | 2E-89 | 263 | prohead core protein (KP15) |
| ORF217 | 215 | 99.53 | 6E-152 | 427 | prohead core and protease (KP27) |
| ORF218 | 262 | 100 | 0 | 512 | prohead core protein (KP15) |
| ORF219 | 522 | 99.23 | 0 | 1060 | major capsid protein (KP15) |
| ORF220 | 429 | 99.07 | 0 | 878 | capsid vertex protein (KP27) |
| ORF221 | 139 | 100 | 5E-97 | 283 | UvsY (KP15) |
| ORF222 | 58 | 100 | 1E-34 | 119 | Unknown (KP15) |
| ORF223 | 26 |  |  |  | Unknown |
| ORF224 | 77 | 100 | 5E-48 | 154 | DNA helicase (KP15) |
| ORF225 | 499 | 100 | 0 | 1040 | helicase (KP27) |
| ORF226 | 192 | 96.35 | 2E-137 | 389 | minor capsid protein inhibitor of protease (KP27) |
| ORF227 | 103 | 99.03 | 4E-71 | 214 | Unknown (KP15) |
| ORF228 | 189 | 99.47 | 3E-138 | 391 | Unknown (KP15) |
| ORF229 | 353 | 40.38 | 2E-34 | 143 | whisker protein (Escherichia phage Lw1) |
| ORF230 | 91 | 98.9 | 2E-60 | 186 | Hoc large outer capsid protein (KP15) |
| ORF231 | 172 | 99.42 | 3E-125 | 357 | Unknown (KP27) |
| ORF232 | 154 | 100 | 2E-108 | 313 | Unknown (KP15) |
| ORF233 | 279 | 99.64 | 0 | 567 | Unknown (KP15) |
| ORF234 | 291 | 93.47 | 0 | 572 | Unknown (KP15) |
| ORF235 | 105 | 99.05 | 1E-72 | 218 | Unknown (KP15) |
| ORF236 | 58 | 94.83 | 1E-31 | 111 | Unknown (KP15) |
| ORF237 | 108 | 99.07 | 9E-73 | 219 | Unknown (KP15) |
| ORF238 | 118 | 100 | 1E-80 | 239 | Unknown (KP15) |
| ORF239 | 174 | 100 | 1E-126 | 360 | postulated decoy of host sigma70 or sigmaS (KP27) |
| ORF240 | 339 | 99.71 | 0 | 699 | rnlB, RNA ligase 2 (KP27) |
| ORF241 | 193 | 98.45 | 1E-138 | 392 | Unknown (KP15) |
| ORF242 | 174 | 97.7 | 1E-123 | 353 | Unknown (KP15) |
| ORF243 | 86 | 98.84 | 3E-55 | 172 | Unknown (KP27) |
| ORF244 | 149 | 98.66 | 1E-104 | 303 | Unknown (KP27) |
| ORF245 | 477 | 100 | 0 | 991 | nicotinamide phosphoribosyl transferase (KP15) |
| ORF246 | 100 | 99 | 1E-66 | 203 | Unknown (KP27) |
| ORF247 | 283 | 98.94 | 0 | 583 | Dam, DNA adenine methylase (KP15) |
| ORF248 | 91 | 100 | 4E-62 | 191 | NrdC, thioredoxin (KP15) |
| ORF249 | 160 | 100 | 2E-117 | 336 | EndoVII, recombination endonuclease VII (KP15) |
| ORF250 | 707 | 99.43 | 0 | 1478 | NrdD, anaerobic ribonucleotide reductase subunit (KP15) |
| ORF251 | 80 | 98.75 | 4E-51 | 162 | Unknown (KP27) |
| ORF252 | 164 | 97.56 | 5E-114 | 328 | Unknown (KP15) |
| ORF253 | 68 | 100 | 1E-42 | 139 | Unknown (KP15) |
| ORF254 | 170 | 98.82 | 3E-121 | 346 | Unknown (KP15) |
| ORF255 | 83 | 98.8 | 6E-56 | 174 | Unknown (KP27) |
| ORF256 | 64 | 100 | 5E-39 | 130 | Unknown (KP15) |
| ORF257 | 175 | 98.29 | 9E-122 | 348 | Unknown (KP15) |
| ORF258 | 163 | 99.39 | 2E-117 | 336 | NrdG, anaerobic nucleotide reductase subunit (KP15) |
| ORF259 | 319 | 93.73 | 0 | 597 | Unknown (KP15) |
| ORF260 | 222 | 98.2 | 3E-157 | 442 | Unknown (KP15) |
| ORF261 | 93 | 100 | 8E-63 | 192 | glutaredoxin (KP27) |
| ORF262 | 99 | 98.99 | 4E-67 | 204 | Unknown (KP15) |
| ORF263 | 1257 | 98.57 | 0 | 2526 | long tail fiber proximal subunit (KP15) |
| ORF264 | 374 | 98.93 | 0 | 763 | hinge connector of long tail fiber proximal connector (KP27) |
| ORF265 | 222 | 99.55 | 8E-159 | 446 | hinge connector of long tail fiber distal connector (KP27) |
| ORF266 | 1394 | 75.96 | 0 | 2049 | L-shaped tail fiber protein (KP15) |
| ORF267 | 175 | 39.01 | 6E-23 | 97.4 | distal long tail fiber assembly catalyst (vB_CsaM_GAP161) |
| ORF268 | 215 | 99.53 | 2E-159 | 446 | T holin (KP15) |
| ORF269 | 89 | 100 | 7E-61 | 187 | Unknown (KP15) |
| ORF270 | 96 | 97.92 | 2E-65 | 199 | Unknown (KP15) |
| ORF271 | 265 | 99.25 | 0 | 545 | Unknown (KP27) |
| ORF272 | 75 | 98.67 | 1E-49 | 158 | Unknown (KP27) |
| ORF273 | 110 | 99.09 | 1E-75 | 228 | Unknown (KP15) |
| ORF274 | 44 | 95.45 | 5E-22 | 86.3 | Unknown (RB16) |
| ORF275 | 82 | 100 | 2E-50 | 160 | Unknown (KP15) |
| ORF276 | 145 | 100 | 2E-104 | 301 | Ndd, nucleoid disruption protein (KP15) |
| ORF277 | 124 | 97.58 | 6E-83 | 246 | Unknown (KP15) |
| ORF278 | 296 | 99.32 | 0 | 616 | rIIB (KP27) |
